# Using Gesture Recognition for AGV Control: Preliminary Research

**DOI:** 10.3390/s23063109

**Published:** 2023-03-14

**Authors:** Sebastian Budzan, Roman Wyżgolik, Marek Kciuk, Krystian Kulik, Radosław Masłowski, Wojciech Ptasiński, Oskar Szkurłat, Mateusz Szwedka, Łukasz Woźniak

**Affiliations:** 1 Department of Measurements and Control Systems, Silesian University of Technology, Akademicka 10A, 44-100 Gliwice, Poland; 2 Department of Mechatronics, Silesian University of Technology, Akademicka 10A, 44-100 Gliwice, Poland

**Keywords:** gesture recognition, neural networks, automatic guided vehicle, HMI

## Abstract

In this paper, we present our investigation of the 2D Hand Gesture Recognition (HGR) which may be suitable for the control of the Automated Guided Vehicle (AGV). In real conditions, we deal with, among others, a complex background, changing lighting conditions, and different distances of the operator from the AGV. For this reason, in the article, we describe the database of 2D images created during the research. We tested classic algorithms and modified them by us ResNet50 and MobileNetV2 which were retrained partially using the transfer learning approach, as well as proposed a simple and effective Convolutional Neural Network (CNN). As part of our work, we used a closed engineering environment for rapid prototyping of vision algorithms, i.e., Adaptive Vision Studio (AVS), currently Zebra Aurora Vision, as well as an open Python programming environment. In addition, we shortly discuss the results of preliminary work on 3D HGR, which seems to be very promising for future work. The results show that, in our case, from the point of view of implementing the gesture recognition methods in AGVs, better results may be expected for RGB images than grayscale ones. Also using 3D imaging and a depth map may give better results.

## 1. Introduction

Gesture recognition in general refers to recognizing the expression of motion by a human, mainly hands and arms but also face, head or even the whole body. Gestures can be, and in many situations already are, an excellent means of interaction between human and human or human and machine. For example, in [1] authors describe a gesture recognition system for interaction with a computer, defining the gestures for mouse movement and keyboard arrow press/depress. For smart home automation, the Wi-Fi based gesture recognition is investigated, based on channel state information (CSI) [2,3]. With CSI the micro human body movement can be detected in a non-intrusive manner, cause human body movement, so also the gestures, would interface the Wi-Fi signal propagation, which is observed as variations of CSI amplitude and phase.

In recent years, there has been a dynamic development in vision technology used for the location and mapping of the AGV, also of the AMR (Autonomous Mobile Robot) environment, the detection of obstacles and people, as well as human-machine collaboration. Gesture control is already used in many civilian applications, in particular related to the automotive industry, e.g., control of infotainment systems or cooperation with collaborative robots, where the main tasks are pick and place, palletizing, packaging, or quality inspection. The main purpose of using AGVs, and now more often collaborative robots in combination with a vehicle, is cooperation with people in the production process, e.g., the robot uses elements that are given to it by a human, performs tasks with elements of significant mass. Of course, in this situation, there is a problem with ensuring safety while increasing production efficiency. Then we must use systems that will allow cooperation in one work zone shared by a human and a vehicle/robot, or ensure the separation of these zones. Subsequently, software support should be provided for the detection of dangerous situations, which in the absence of reaction will lead to a collision, then for the collision itself, as well as for determining its level. Initially, the operation of the system was reduced to optical devices that only controlled the appearance of an object—a human figure; in a specific zone of the robot, or vehicle. In recent years, a number of studies and engineering applications have enabled more precise oper ation by introducing human interaction and controlling the robot/vehicle using gestures.

The autonomy of AGVs is constantly being developed. There are solutions on the market, for which the routes do not need to be determined by wires embedded in the floor, coloured or magnetic tapes applied to the floor surface. They use laser scanners to create a map of their surroundings and use SLAM (Simultaneous Localization and Mapping) system for localization and navigation. The advantage is that the map is continuously updated during the AMR movement/driving. Due to the location of the laser scanner in AMR, they create a map of objects in the surrounding up to a certain height, usually 20–30 cm. Therefore, e.g., obstacles hanging above the floor at a height above the laser range may pose a problem. Therefore, AMR vehicles are usually equipped with one or two 2D cameras to detect such objects. The cameras can be used to extend AMR functionality with gesture recognition. For example, gesture recognition can be used to call the nearest free AMR vehicle and stop it at a certain distance from the operator, it can also be used when parking a vehicle in working zones at production lines, docking in loading zones, moving between halls. Of course, in zones where vehicles move in an environment shared with employees. There, gesture control can be useful, acting much more selectively than typical security systems based on 2–3 zones determined in a laser scanner. Due to the need to ensure the speed and efficiency of software operation, it is so important to work on the weaknesses of gesture detection algorithms in industrial applications.

Hand gesture recognition in automotive human-machine interaction (HMI) is also a highly focused area of research. Ref. [4] gives an overview of early HMI trends for human-vehicle interaction with more than 40 references concerning the topic. Also a number of technics for hand gesture interface can be found there. The current approach to hand gesture recognition for automotive applications is discussed in [5], where authors propose a system with an infrared array sensor. The advantage of the infrared approach is that it can work in various conditions as day and night, in noisy environment and in tunnels. The alternative approach, based on depth cameras, is presented in [6]. The authors describe current Machine Learning approaches to hand gesture recognition with data from depth cameras.

In [7] authors propose a system for guiding unmanned vehicle based on gesture recognition. The vehicle recognizes the “follow” and “stop” hand gestures shown by a person. The first one puts the vehicle into follow (guide) mode so the vehicle begins to detect the person then track and follow it. To finish guiding, the person issues a “stop” gesture. The sensor used in this work was the Kinect-V2.

For robot applications in [8] the system is proposed based on PDM Camboar Pico ToF camera which, according to the authors, gives 100 better accuracy than the stereo camera. However, the range of the device used is limited to 4 m, which is sufficient in the case of the application described there.

Since 2013/14, we have seen a significant increase in publications dealing with gesture recognition. The most recent publication status regarding gesture recognition can be found in [9]. This study has analysed 571 papers related to artificial intelligence and gesture recognition. We can see, that deep learning (DL) methods are the most popular, however also hybrid methods are used in some cases, e.g., for continuous gesture recognition on data comprising image and depth data and skeleton features [10] with HMM and deep dynamic neural networks (DDNN) or [11] for hand gesture recognition with a combination of 3D CNN and a support vector machine (SVM) classifier.

One of the first survey on gesture recognition, with particular emphasis on hand gesture and face expressions, has been published by Mitra and Acharya [12] with 64 referenced papers. It focuses on different aspects of gesture recognition. Most of the problems have been addressed based on statical modeling, such as principal component analysis, hidden Markov models (HMM), Kalman or particle filtering. The currently widely used neural networks were then represented by multilayer perceptron (MLP) and time delay neural network (TDNN). 2007 was the year of the initial release of NVIDIA CUDA technology for GPU processing, making it possible to use more complex neural networks, in following years, such as long short-term memory (LSTM) [13,14] or convolutional neural networks (CNN) [15,16].

Due to their specificity and frequency of use, it is hand gestures that are most often recognized. For the rest, sign language uses a set of hand gestures for a reason—we can convey more information with our hands than facial expressions or posture. Hand modeling and 3D motion based pose estimation methods are reviewed in [17] and cover developments till the year 2005, according to the listed references. The review of vision-based hand gesture recognition algorithms is given in [18]. However this is also not the newest publication, it is focused strictly on hand gestures. The major challenge is the identification of the hand gesturing phase in an automatic gesture recognizer. First, we have to identify the hand in the complex scene, then detect the known gesture from the unpredictable and ambiguous non-gesture hand motions. The poorly lit scene, background colour close to the colour of the skin, the distance between the camera and the human, scene complexity (many elements in the scene) are the problems that determine the reliability of the detection algorithm. Especially the last two are important from the point of view of hand gesture recognition applications in use for AGVs. The problem addressing issues due to complex backgrounds is discussed e.g., in [19,20]. The threshold model concept, using HMM, is discussed in [21] and simultaneous gesture segmentation and recognition is proposed in [22].

If we take into account the taxonomy of gestures, there are multiple ways to categorize hand gestures. The categorization is given by Pishardy, Saerbek in [18]. First, based on observable features. Second, based on the interpretation. In this paper, we focused only on the first from above, where gestures are classified, based on temporal relationships into two types: static and dynamic. In static gesture (aka hand postures/poses) we are looking for orientation, shape, finger’s flex angles, relative position to body and/or context environment. Dynamic gestures are characterized, besides the shape, orientation, and finger’s flex angles, with position/trajectory, motions speed/direction and scale.

In this work, we described an approach to recognizing selected gestures in 2D images for the purposes of controlling an autonomous platform. The main contributions of our paper are summarized as follows:

- We have developed a diverse database of ten gestures with a complex background, changing lighting conditions, and different distances of the operator from the AGV. Our image database also contains clipped gestures.
- We proposed a straightforward and effective simple CNN that handles RGB images well and does not require a large number of iterations to train.
- We modified the pre-trained ResNet50 and MobileNetV2 networks for the problem of 2D and 3D gesture recognition.
- We modified and implemented convexity defects algorithm in AVS and Python environment.
- We conducted an analysis of the obtained results, generated by convexity defects detection and neural networks, in terms of incorrect classification of gestures, so as to indicate among them those gestures that will ensure high reliability of classification.
- We conducted a valuable comparison between a AVS engineering software, and an Python environment, pointing out the pros and cons in terms of creating a neural network structure.
- We conducted a preliminary analysis of the possibility of using neural networks for gesture recognition in 3D images.

The rest of the paper is organized as follows. In Section 2 we described the equipment utilized to acquire the images, the images database for training the neural networks. Then the problems connected with gesture recognition in 2D and 3D images and the software tools selected for research. In Section 3 we provide a discussion of the results for conventional gesture recognition methods based on image segmentation and morphology and the deep learning methods for 2D and 3D images using customized CNN networks and implemented in AVS. At the end, in Section 4 we summarize our research and provide the conclusions.

## 2. Materials

### 2.1. Hardware

**Figure 1:**
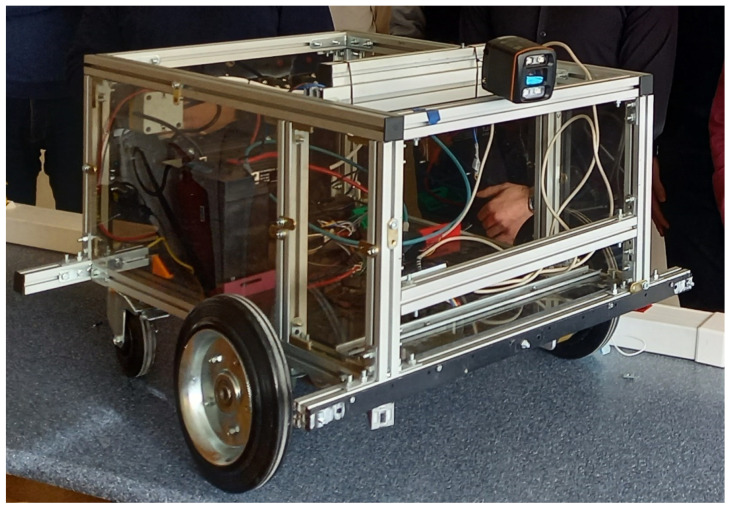
Experimental AGV platform.

As part of our work, we use the prototype AGV platform developed in the Department (Figure 1), which has been equipped with a power supply, drive, control system, and a mapping, location, and object detection system. The drive of the platform consists of two wheels located in the front part of the chassis and a single swivel wheel located in the rear part of the chassis. From the point of view of the drive system of the platform, each of the two front wheels is coupled to an independent DC motor. The additional rear wheel of the vehicle allows free rotation in the axis normal to the plane of the platform’s motion, and thus enables to change the direction of the vehicle. The vehicle control system was organized based on the open-source Robot Operating System (ROS) programming platform. The mapping, location and object detection system works with the use of 2D scanners, vision cameras, and distance sensors.

Gesture detection was performed using Basler 2D camera acA1600-20gc with 1624 × 1234 resolution, 20 frames per second, and 8 mm lens, also with an additional 3D IFM camera O3D302 with 176 × 132 resolution and 300–8000 mm working distance.

### 2.2. Gesture Images Database

The starting point in our research was the most effective linking of specific gestures with unambiguous behavior of the vehicle, i.e.: starting/stopping the vehicle, driving straight, turn left, turn right. For this reason, in our considerations, we focused on 10 gestures that were recorded in visible light using a 2D camera as the main basis used in our research. On the other hand, the registered—smaller, database of 3D images served us only as a potential development path in the future, both in terms of the advantage over 2D image acquisition and a number of classification algorithms that are used to detect gestures in 3D space or the depth map.

**Figure 2:**
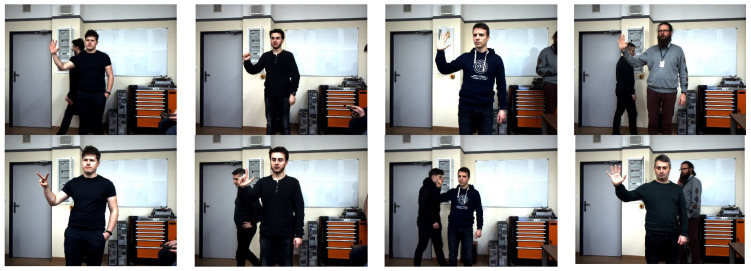
Sample raw 2D images taken during experiments.

The images were recorded in natural indoor conditions. During the recording, disturbances typical for AGV/AMR applications in the form of various backgrounds (uniform, complex, mixed—part of the hand was on a uniform background, remained on a complex one), variable lighting, and additional human poses in the background were taken into account. During the registration, features related to image acquisition, i.e., loss of sharpness, overexposure of the matrix, were also taken into account. The diversity of the base was also ensured, i.e., 6 people of different ages and different clothes were used. Images were recorded from two different distances—1.0 and 2.5 m. Each gesture was performed from multiple angles by each study participant independently, resulting in high variability within classes by background, scale, shape, m, and gesture angle. Sample images have been presented on Figure 2.

**Figure 3:**
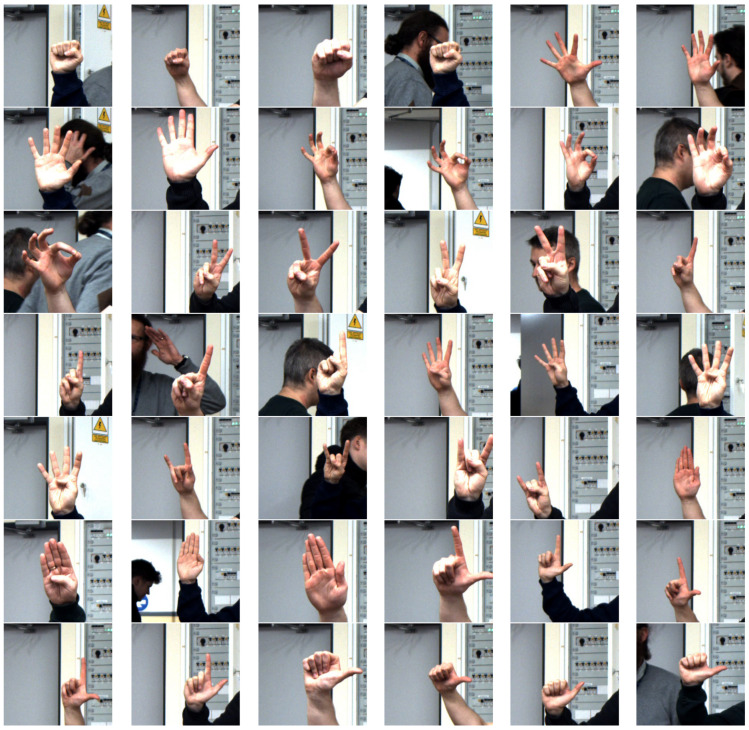
2D sample images of gestures from left to right: Fist(0), Palm(1), Rabbit(2), Victoria(3), One(4), Four(5), Rock(6), Stop(7), Loser(8), Thumbleft(9).

Ten gestures were randomly selected for the study, namely: Fist(0), Palm(1), Rabbit(2), Victoria(3), One(4), Four(5), Rock(6), Stop(7), Loser(8), Thunderbolt(9). The recorded images originally had a resolution of 1624 × 1234 and contained a full scene with human poses and complex surroundings. The database contains 4750 images in total -475 images per gesture (Figure 3). The hand area in the original images averages 150x150 pixels. For this reason, in all algorithms, we decided to analyze only the image from the region of interest around the hand. Regardless of the tested algorithm, the input images had a resolution of 282 × 266, which was determined based on the Region Of Interests (ROI) algorithm, based on the conversion of the RGB space to HSV and the extraction of the H component. interest in the hand. In the article, we also show the disadvantages of this method in practice, which are revealed especially when a human head or another hand appears in the background. The distance from the camera, other silhouettes in the background in this situation only result in a change of position—centering the hand relative to the ROI, and in critical cases even an incomplete shape of the hand in the ROI. In our considerations, we have taken into account all the listed situations and features.

The use of 2D images for gesture recognition has a major disadvantage—the person showing the gesture must be relatively close to the camera to reduce the impact of the aforementioned disturbances, especially in industrial conditions. The image obtained with a 2D camera also does not provide information about the distance of objects, therefore the analysis of the image with a larger number of people significantly complicates the algorithm or requires additional restrictions imposed on the operator in terms of the method—direction, position, showing a gesture in front of the vehicle. Also for the above reason, research is also carried out in the field of gesture detection in images obtained from 3D cameras.

**Figure 4:**
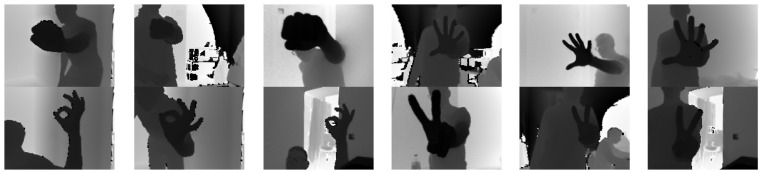
Depth images of gestures from left to right: Fist(0), Palm(1), Rabbit(2), Victoria(3).

The 3D camera used in the project was a time-of-flight camera, in which the coordinates of points are calculated based on the measurement of the time of flight of the beam sent by the generator. The result is a 3D point cloud. Analyzing raw 3D point clouds involves significant computational effort, so it was limited to a 2D image while preserving distance information through the use of a depth map. It is created by projecting each point of the 3D point cloud onto the sensor plane and assigning weights in the range 0–255 depending on the distance of the respective points. Sample depth maps are shown in Figure 4.

There are about 950 images for each of the four classes, they were acquired with resolution 176 × 132 pixels from different distances, angles, with changing lighting, and other people in the scene. The article describes depth map processing only as a direction of potential development of human interaction systems with AGVs using gestures, therefore the database currently contains only four gestures and the results were not directly compared with the results obtained based on 2D images in visible light.

### 2.3. Software Background

Due to the practical application of the gesture detection algorithm, the experiments were conducted from the scientific side in terms of potential, effective detection methods, and from the implementation side of various programming environments. For this reason, among the methods, we implemented the gesture detection method based on the search for convexity defects, the ResNet50 and MobilnetV2 deep learning method modified by us, as well as we proposed a simple and fast CNN network. We compared the algorithms implemented in the Python environment with the results obtained in a typical engineering environment for image processing, which is Adaptive Vision Studio, currently Zebra Aurora Vision Studio. The environment is easy to use for both maintenance engineers and scientists dealing with the subject of machine vision, object detection and classification. At the same time, it has a wide range of tools for vision control. As part of the classic algorithm, we used basic functions in the field of image segmentation and morphology, while the neural network learning process required the use of the Deep Learning tool, which allows for a transparent preparation of the learning process, including setting the learning parameters, including data augmentation. This tool does not require network structure design, but on the other hand, we also have no way to know its structure. The Deep learning module uses deep neural networks, using Transfer Learning, in which a pre-trained network is reconfigured at some stage to adapt to the current problem.

## 3. Methods, Discussion and Results

**Figure 5:**
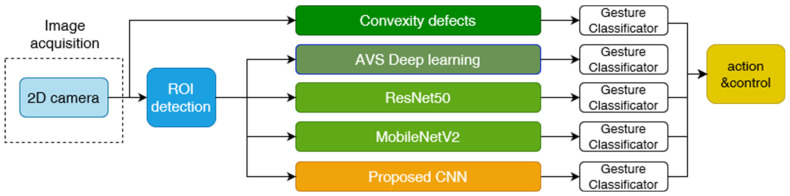
Flow diagram of 2D image processing.

In this section, in the following subsections, we presented the detailed description, discussion and results obtained for the method based on convexity defects recognition known from the literature, then modified by us methods of deep learning of 2D images ResNet50 and MobileNetV2, and the simple and fast CNN network proposed by us. The discussion was focused on the analysis of mainly the disadvantages of the methods due to the accuracy of detection and recognition of gestures. In addition, we conducted preliminary studies of gesture detection based on a 3D camera and a depth map. In this regard, we also tested selected neural networks. A summary of the 2D experiments concept is presented in Figure 5. The above solutions were developed in the Python environment, while the solution based on image segmentation and neural networks was also additionally designed in the Adaptive Vision environment.

### 3.1. 2D Gesture Recognition Based on Convexity Defects

The first approach was to use classical methods, which are most often based on the extraction of specific features of the shape of the object. A common approach in the classification of hand gestures is to extract the area based on the differences between the colour of the hand and the environment [23,24]. The aim is therefore to search for characteristic points, called convexity defects [25], which should be detected in the areas between the fingers. In practice, these are the points of the hand contour that are furthest away from the convex polygon surrounding the hand.

**Figure 6:**
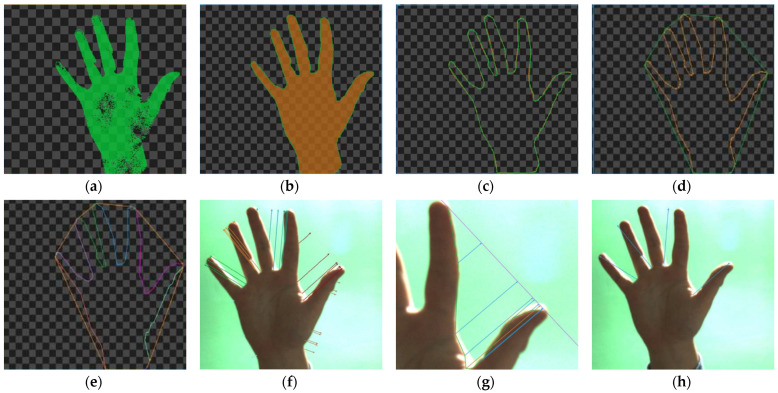
Image of extracted hand area (**a**), hand after morphological closing (**b**), contour of the hand (**c**), convex polygon (**d**), segmented contour (**e**), candidates (**f**), longest candidate searching (**g**), final fingers selection—blue lines (**h**).

The method primarily uses segmentation in the HSV space and morphological operations to improve the quality of the detected hand area. Figure 6 shows the results of the most important steps of the convexity defect detection algorithm. First, the image from the RGB space should be converted to HSV, and then the Hue component (Figure 6a) is extracted, this operation allows the skin area to be extracted from the image. Despite the change in space, the original image usually contains much more separated objects than just areas of human skin. Since the effectiveness of classical methods depends primarily on the quality of the palm area, all other objects should be reduced. We used area filtration, which allowed us to reduce smaller, individual areas on the one hand, and the remaining areas are reduced based on the calculated area. Then, the image is improved on the basis of morphological operations, mainly closing with a 5 × 5 kernel, which allows to fill any holes in the hand area (Figure 6b). In the next step (Figure 6c), the contour was returned using the topological structural analysis algorithm [26] in our python implementation and *RegionContours* filter in AVS software respectively. In Figure 6d convex polygon of the hand area is presented, which is used to divide the contour of the hand into segments, which are presented in Figure 6e in the form of different colours.

The contour separated in this way is used to determine the shortest segments connecting the points of the segments with the corresponding side of the polygon. The result are groups of segments that are candidates representing fingers (Figure 6f). The last step is to reduce the number of candidates (Figure 6g) by searching for the longest segment in each group. At this stage, the selected candidates are also verified by checking the value of the angle between neighboring candidates—it must be less than 90° (Figure 6h).

**Figure 7:**
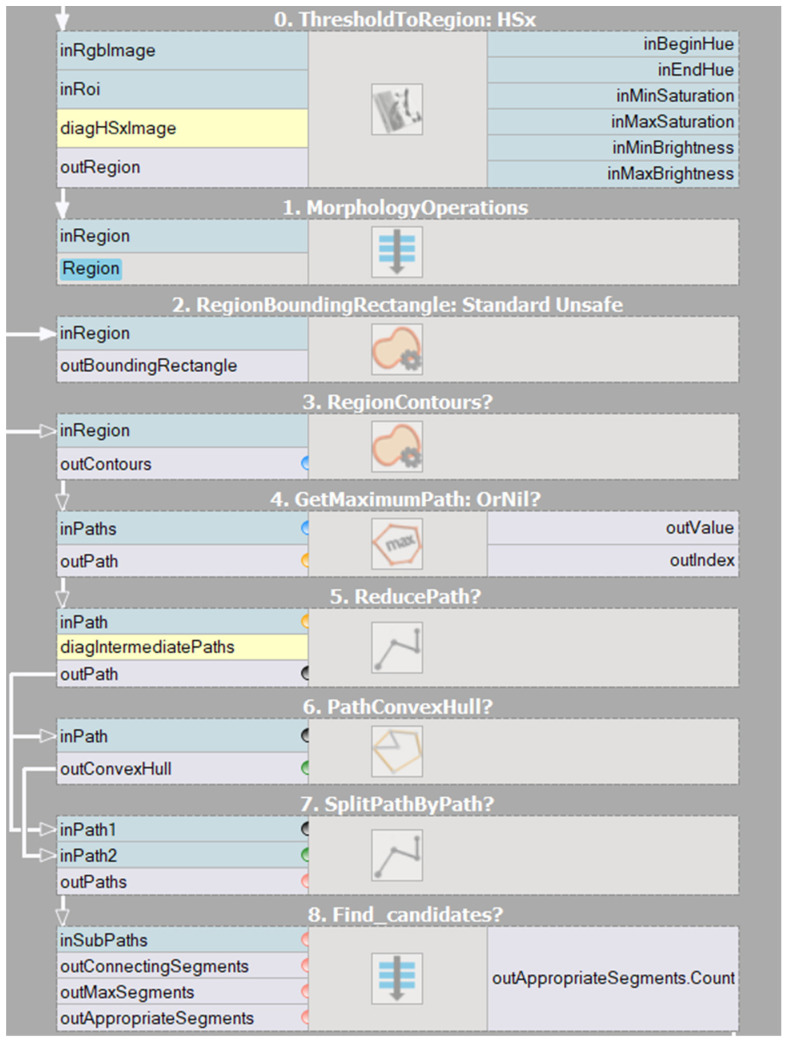
Developed hand gesture algorithm diagram in AVS software.

Figure 7 presents a similar algorithm developed during our experiments in AVS containing the entire algorithm. Individual parts of the program have been divided into sections, namely thresholding, morphological operations, determination of the hand contour, contour reduction, determination of the envelope of the hand, and determination of characteristic points.

**Figure 8:**
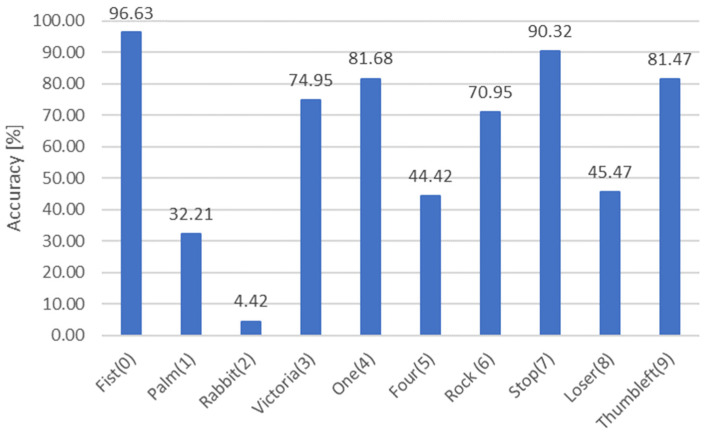
Accuracy for all the gesture’s classes.

In principle, the algorithm should allow for an unambiguous determination of the gesture by estimating the number of convexity defects—the space between the fingers. However, this conclusion is true only for images obtained in conditions ensuring high contrast in the image. Using the 2D image database described in the previous section, the following results of convexity defects detection were obtained (Table 1). Bold numbers indicate the number of defects that should be obtained. The results show that the classical method has significant problems with the detection of convexity defects. 4750 images were tested, if only the global detection accuracy for ten gestures was taken into account, it is 62.25%. On the other hand, the analysis of the results for individual gesture classes is more important due to practical application, on the one hand, the accuracy of the detection of a single gesture class, and on the other, the possibility of classifying a gesture among ten classes. A summary of the calculated accuracies is presented in Figure 8.

For two gestures Fist(0), Stop(7) the accuracy was above 90%, for the next two gestures One(4), Thumbleft(9) above 80%. A characteristic feature of these four gestures is the expected number of convexity defects—0. The worst results were obtained for two gestures, Rabbit(2), Palm(1), respectively 4.42% and 32.21%. For both gestures, the position of the hand in relation to the camera and the algorithm, which is ultimately based on the number of convexity defects, undoubtedly has a major impact on the detection result. In the case of the Palm(1) gesture, the width of the palm spread is also important, which directly affects the analysis already at the stage of changing the colour space from RGB to HSV. Importantly, all the cases where poor results were obtained were characterized by the detection of several convexity defects instead of one standard one. In this case, there is the Rabbit(2) gesture, which combines two potential disadvantages—the need to spread three fingers wide and the correct position relative to the camera. For this reason, only 21 images contained the correct number of bulge defects (3), and the rest were distributed between 0–2 defects more or less evenly. Among the gestures below 80% detection accuracy, the causes of errors in detection should always be looked for in the incorrect position of the hand, which results in a change in the opening angle between the segment lines (fingers)—a prime example is the Loser(8) gesture, where the algorithm most often does not detect a convexity defect, or in the second place only detects one. In this situation, the most important is the angle between the thumb and index finger. In conclusion, the algorithm fails in every gesture in which there is a problem with unambiguously determining the defect of the convexity between the fingers.

**Figure 9:**
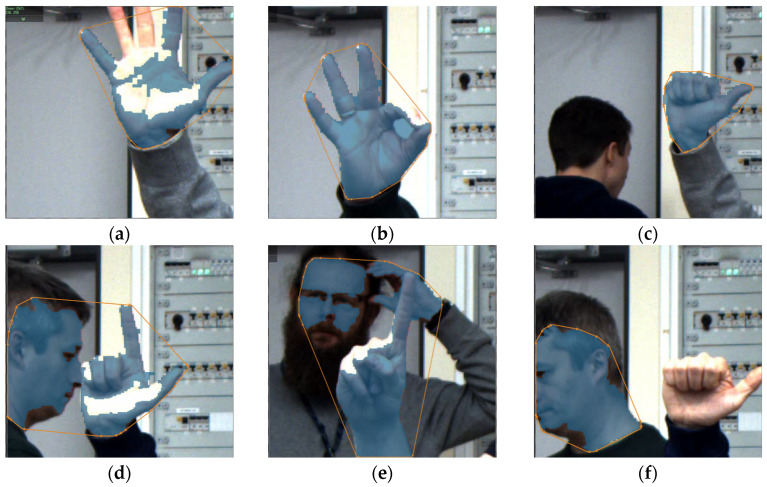
Images of different gestures and faults.

Despite the definitely fast prototyping of the algorithm in the AVS environment, also Python, the method requires, on the one hand, very good image quality—the problem with the thresholding operation, but also the selection of appropriate gestures. As a consequence of the above considerations, in real, industrial conditions, certain restrictions should be introduced, mainly at the stage of image recording, i.e., determine the area of the image in which the gesture should appear and ensure the appropriate distance of the hand from the camera, which will ensure correct focus on hand and sharp hand region in the image. In addition to the reasons related to the preservation of the angle, the shape of the gesture during registration in the classical method, we are dealing with errors resulting from the adopted algorithm based on the HSV space and morphological operations. Figure 9 presents selected problems arising during the experiments. Placing the hand at an angle that causes the reflection of the LED light results in errors in HSV segmentation (Figure 9a), incorrect operation of the morphology operation results in closing too large an area between the thumb and forefinger (Figure 9b) while reflecting the light. In Figure 9c, the shape of the convex polygon has been changed in the thumb area by attaching a fragment of the background object to the thumb area. The Figure 9d–f show problems when there are other skin areas in the scene. In the extreme case (Figure 9f), the palm area may be omitted from the analysis. Equally important as the ability to detect an individual gesture is the ability to classify gestures from several classes, and here, unfortunately, in this case, the method based only on convexity defects does not allow to distinguish gestures for which the standard number of defects is the same.

From the point of view of real-time control of AGV/AMR using gestures, the above-described disadvantages of the classical method, both resulting from the acquisition process itself and image processing, or the unambiguity in the gesture classification, make it necessary to look for methods resistant to the above factors. In industrial conditions, certain restrictions should be introduced, mainly at the stage of image recording, such as limiting the distance and position relative to the camera, even the area of the ROI image where the gesture should appear. For this reason, at the base registration stage, we conducted experiments with forcing the position and ROI area in which the gesture appeared. This ultimately resulted in many images containing incomplete, clipped gestures, blurred gestures as a result of a quick attempt to stabilize the gesture inside the ROI. Regardless of the possibility of introducing restrictions during registration, optimizing classical methods in terms of the processing algorithm, or limiting the number of gestures to those most unique to the algorithm, deep learning methods seem to be an alternative that should cope better.

### 3.2. 2D Gesture Recognition Based on CNN in AVS

**Figure 10:**
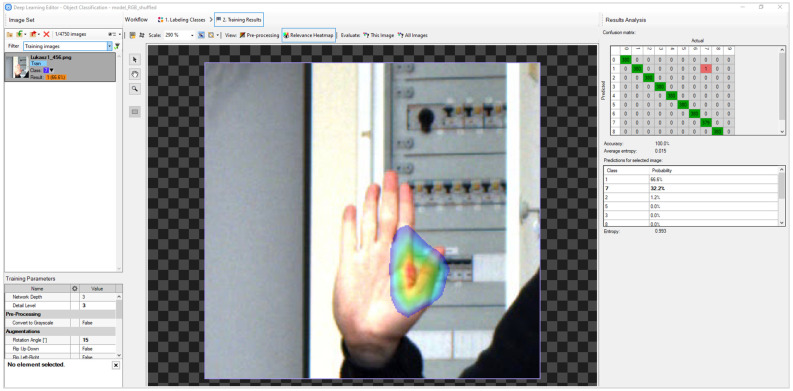
Deep Learning Editor interface in AVS.

In the AVS environment, the Classify Object deep-learning tool was used in a uniform way for grayscale and RGB images. Figure 10 shows one of the interfaces from Deep Learning Module.

What is very important, when using the Deep Learning Module tool in AVS, it is not possible to design the network, and it is based on predefined networks with an unknown structure. However, the learning process can be controlled primarily by changing the augmentation parameters, i.e., rotation angle (15°), relative translation (5%), scale of the image (95–105%), noise (2%) and luminance (8%). The same augmentation was used in the other networks. After experimenting with the number of epochs, we finally used 100 epochs for which we obtained optimal results for the tested set of images.

In addition to the direct access augmentation parameters, another advantage is the ability to observe images after passing through the appropriate convolutional layers. The tool is used to determine whether the network is focused on the relevant areas. The image with gesture presented in Figure 10 shows an example of the heatmaps for selected gestures in grayscale images CNN. The area in red is the area that has the most significant impact on assigning a gesture to one of the classes.

Without a doubt, the detection of gestures in the optimal situation should be carried out on binary images. The solution based on binary images definitely has many advantages—short time of classification, training and the test set does not have to be very large. A definite advantage of using binary images is the possibility of creating a very small set. Nevertheless, this approach is not without its drawbacks. The key disadvantage of this approach is being dependent on lighting conditions and implementing additional thresholding and segmentation algorithms. For this reason, despite the advantages, we do not recommend relying on binary images in real, industrial conditions, where most often images contain a complex background that significantly affects the effectiveness of gesture detection. Therefore, we used both grayscale and RGB images in our experiments to explore the potential effect of colour on gesture classification.

**Figure 11:**
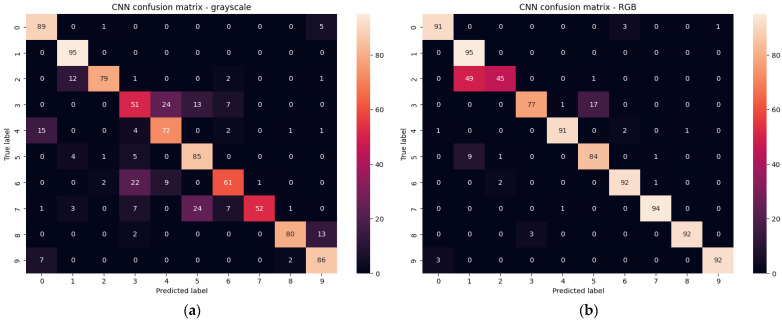
Confusion matrixes for test set of grayscale (**a**) and RGB (**b**) images.

The use of grayscale and RGB images requires a much larger set of images. The number of training and validation images per class was 380 divided proportional 80:20. The parameter that gives important information about the quality of learning is, of course, entropy, which should be interpreted as the uncertainty of the occurrence of a given elementary event. Entropy tending to zero is equivalent to a stronger classifier. In the training process, entropy of 0.022 and 0.015 was obtained for grayscale and RGB images, respectively. Accuracy of 99.95% (grayscale) and 99.97% (RGB) was obtained for 3800 training images of all classes. The test was performed on a set of 950 of all gesture classes that were recorded as a separate set of images in the same environment, but none of these images were in the training set. The same set of test images was used to test the rest of the networks. The result was an accuracy of 78.95% (200 grayscale images misclassified) and 89.79% (97 RGB images misclassified). The detailed distribution of errors is shown in the confusion matrices for grayscale (Figure 11a) and RGB (Figure 11b) images.

Analyzing the results for grayscale images, significant errors in the classification of gestures Victoria(3), Rock(6), Stop(7) are characteristic, reaching even 46%. Gesture combinations that have been particularly confused are Rabbit(2) with Palm(1), Victoria(3) with One(4) and Four(5), One(4) with Fist(0), Rock(6) with Victoria(3), Stop(7) with Four(5) and Loser(8) with Thumbleft(9). The best results were obtained for the Fist(0), Palm(1), Thumbleft(9) gestures, which reached a maximum number of errors of 9.47% for the Thumbleft(9) gesture, and the Palm(1) gesture was recognized without errors.

In the case of using the classifier for RGB images, much better results were obtained. Errors were significantly reduced as eight gestures were detected with a minimum accuracy of 88.42%. In only one Rabbit(2) gesture, a deterioration of accuracy from 83.15% to 47.36% was observed, which for RGB is more than three times more likely to be confused with the Palm(1) gesture. It is a gesture which, due to the highly differentiated hand positioning of the study participants, may be confused with the Palm gesture(1).

**Figure 12:**
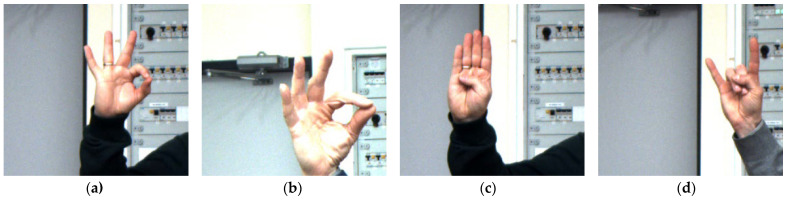
Rabbit(2) classified as Palm(1) (**a**,**b**), Stop(7) as Four(5) (**c**), Rock(6) as Victoria(3) (**d**).

Moreover, the learned classifier for RGB images is much more selective and classifies erroneous detections with greater confidence. Figure 12 shows some representative examples of misclassification. In Figure 12a, the gesture is misclassified with a probability of 80.3% for grayscale versus 98.5% for RGB. For the image in Figure 12b, it was 52.6% versus 98.9% (b). Misclassifications for grayscale images that were completely reduced for RGB were most likely to have high misclassification probabilities—Figure 12c with 93.9% and Figure 12d with 93,8% probability).

The use of closed software such as AVS, which is designed for object detection, can be an alternative to open algorithms, where it will be necessary to quickly prototype a solution for large learning sets in the case of gestures. One has note that in this situation we cannot able to change the structure of the network, which is a significant limitation in optimization of the classification for RGB images of gestures. From the point of view of AGV/AMR control, in this situation, it would be advisable to focus on the selection of only those gestures that not only achieve high detection accuracy, but false detections do not occur between them, for example: Palm(1), Stop(7), Thumbleft(9).

### 3.3. 2D Gesture Recognition Based on Customized CNN

During the experiments, we proposed a developed and parameterized network, implemented in Python using the Keras/Tensorflow library. An important stage in the implementation of the training is the determination of the so-called hyperparameters. Hyperparameters of the network are parameters that are not subject to training, i.e., they are constant and top-down selected. The hyperparameters include, among others: network architecture—types of layers and selection of activation functions, batchsize—the number of sample training data used during one training iteration, epochs—the number of training iterations, loss and learning rate—loss function and learning coefficient, optimizer—tool calculating new weights, number of training, validation and test images, dimensions of input images. The proposed CNN network architecture is presented in Table 2.

All images were uniformly resized to 244 × 244 pixels. In the conducted experiments, the optimal values for each trained network were determined. Finally, the batch size was set to 16. The Adam optimizer was used with default parameters, so the learning rate equals 0.001, beta_1 = 0.9, beta_2 = 0.999. The standard loss function for a multi-class classification problem is set to categorical cross-entropy, which corresponds to the average entropy in the AVS. For all experiments, conducted with our network, we set the same number of iterations for one epoch, equal to 100, and the number of epochs also equal to 100. The kernel size was 5 × 5. All the experiments were performed on NVIDIA GeForce RTX3060 GPU. The *Relu* activation function was used, which resets negative values.

**Figure 13:**
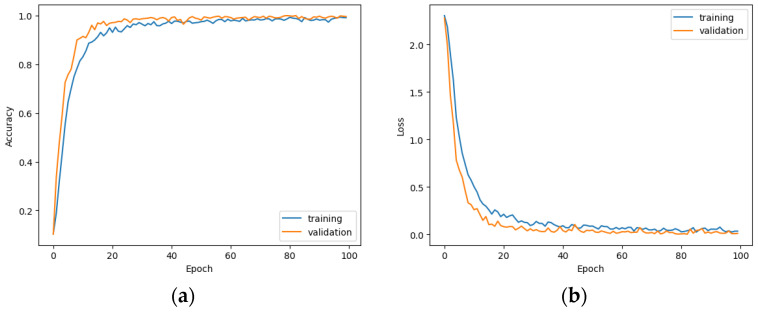
Accuracy (**a**) and loss (**b**) in relation to the number of epochs.

The solutions mentioned above make it possible to create activation maps. Additionally, the so-called dense layer, allowed us to take into account the vector data present in the image (the so-called two-dimensional tensors). Two-dimensional tensors were created after the *Flatten* operation was performed on three-dimensional tensors. This layer was the output layer activated with the *Softmax* function. As in the case of AVS, it was necessary to augment the data to prevent overtraining of the network. The efficiency and loss plots for this model for grayscale images are shown in Figure 13. The accuracy of the model for the test set is 70.5% and the average entropy is 1.72.

**Figure 14:**
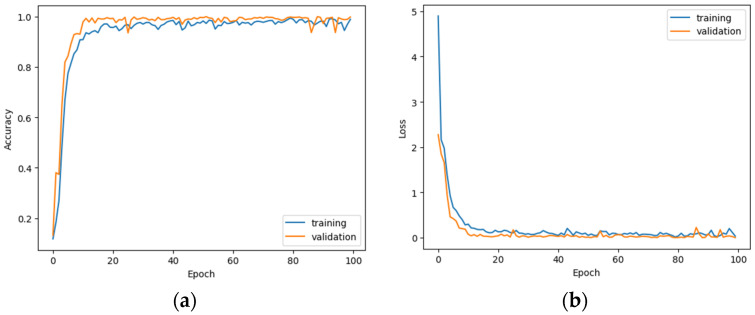
Accuracy (**a**) and loss (**b**) in relation to the number of iterations (epochs).

For RGB images, the network architecture for grayscale images was modified primarily in the input layer—the data from the input images was projected this time to a tensor with dimensions (244, 244, 3). Other hyperparameters remained unchanged. The efficiency and loss plot for this model is presented in Figure 14.

**Figure 15:**
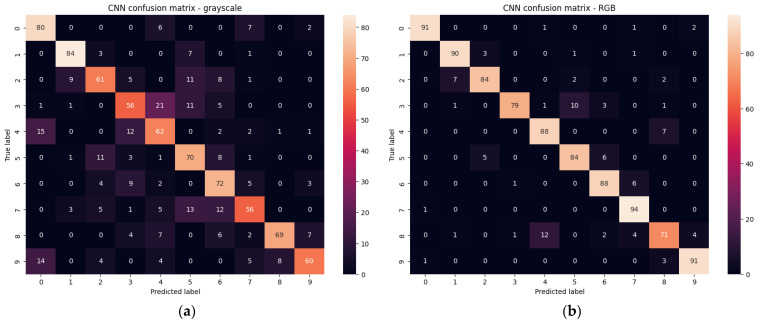
Confusion matrixes for test set of grayscale (**a**) and RGB (**b**) images.

Figure 15 shows the error matrices of the test sets for both, the grayscale and RGB images. For grayscale images, we have quite acceptable recognition, i.e., with a probability of at least 84%, of only two gestures: Fist(0) and Palm(1). Detection of other gestures is at an unsatisfactory level.

On the other hand, the accuracy of RGB model for the training set is 90.53 %, and the worst results were obtained for Looser(8) and Victoria(3) gestures. From the rest of the gestures, we can select for further processing (selecting the gestures for AGV control) these, for which the proper classification is greater e.g., than 89%.

As a validation for our implementation of CNN in Python, we used MobileNetV2 and Resnet50. The MobileNetV2 utilizes depth-wise separable convolutions to build lightweight deep neural networks dedicated mostly to embedded applications [27]. In [28] authors compare recognition performance for hand gesture recognition using MobileNetV3 [29] with the network proposed by the authors and with some other predefined networks such as ResNet101 [30], ShuffleNetV2 [31], and HGR-Net [32]. The results show that the MobileNetV3 has relatively high accuracy, by 3.6% less than the network proposed by the authors. However, there is no information if the authors utilized those networks as is or with a transfer learning approach, except for the last layer for data classification, which has to be adapted to the number of classes. In our investigations, we froze all the layers, except the last 3, which we relearned for our data set. The second version of MobileNet model [33] was utilized, specifically tf2-preview/mobilenet_v2/feature_vector/4 from Tensorflow Hub. The MobileNetV2, as well as ResNet50, are trained on a large dataset so we use a transfer learning approach [34] to adapt the networks to analysed gestures.

For the Resnet50 neural network model, the last 3 layers were removed according to the transfer learning principle. In their place, the two Dense layers with a neuron count of 2048 were added to the model, as well as accompanying Dropout layers, which perform regularisation functions in the learning process. Without the aforementioned regularisation layer, the learning process generated an overlearned model, so the use of dropout was necessary for this purpose. One of the last layers of the model described is BatchNormalisation, a technique that standardises the inputs to the layer for each mini-batch. By attaching this layer to the network model, it is possible to stabilise the learning process and reduce the number of training epochs required to train deep networks. The output of the Resnet50 model includes a layer with ten neurons, corresponding to the number of classes in the classification problem under analysis. Thanks to the softmax function used in this layer as an activation function, the output gives a polynomial probability distribution for each processed gesture.

**Figure 16:**
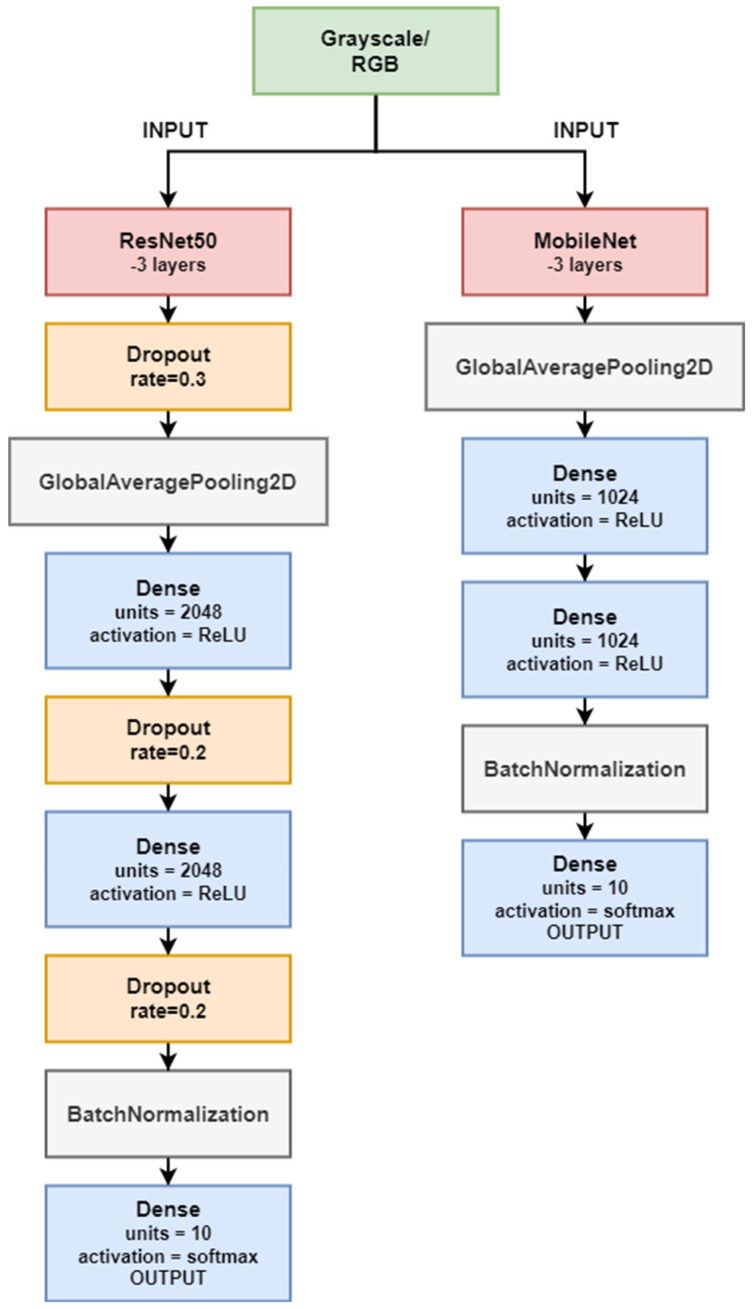
Resnet50 and MobileNet structure. Top blocks representing frozen layers—3; the rest are 3 layers replaced for transfer learning.

With the application of MovilenetV2, the situation is similar to the Resnet50 with ten neurons at the output. The difference between these networks, however, is that the network performed better without additional regularisation functions. Therefore, in this model also as in Resnet50, the last 3 layers were removed, while 2 more Dense layers were added. These layers have fewer neurons than ResNet50. The diagram representing both networks is shown in Figure 16.

**Figure 17:**
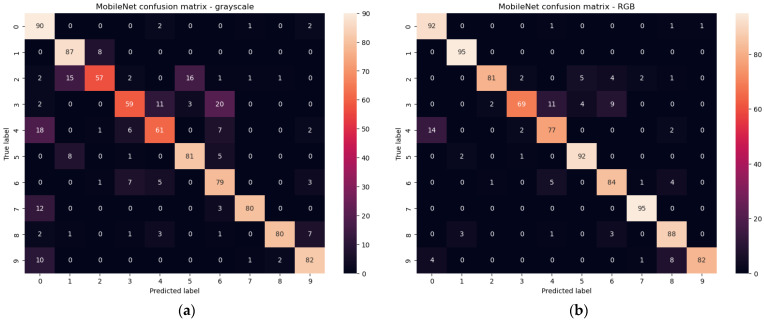
Confusion matrixes for test set of grayscale (**a**) and RGB (**b**) images for MobileNetV2.

With this approach, the MobilenetV2 gave us 78.4% accuracy and 0.3 loss for test set of grayscale images while for RGB images, 90.1% test accuracy and about 0.3 loss. In Figure 17 the confusion matrixes are presented, for grayscale and RGB test sets. The result for RGB images is close to our CNN, but the size of the classifier is larger (see Table 3).

**Figure 18:**
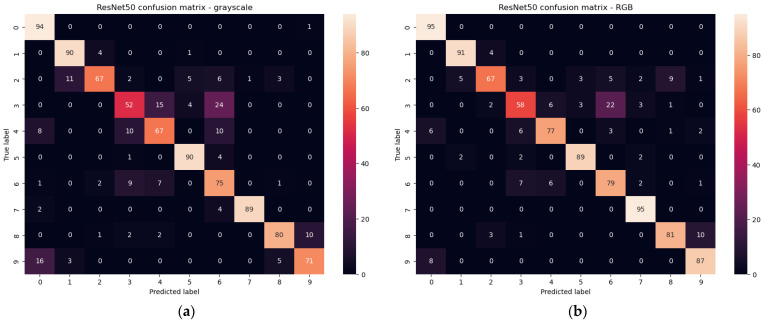
Confusion matrixes for test set of grayscale (**a**) and RGB (**b**) images for ResNet50.

For the RestNet50 [34] we obtained 81.8% accuracy for the grayscale images test set and 85.0% accuracy for RGB images test set. The result for RGB images is rather disappointing, and additionally, the size of the classifier is significant (see Table 3). The confusion matrixes for test sets are presented in Figure 18.

### 3.4. Summary

Table 3 presents information on the results and parameters of the models trained in this work. Train accuracy and Test accuracy are the accuracies on the training and test sets. Train loss and Test loss represent the values of the loss function (average entropy), while Classifier size is the size of the model in megabytes.

As part of the work, the classification of selected gestures in 2D images was investigated. The methods are based on classic solutions using segmentation as well as CNN network including pretrained MobileNetV2 and ResNet50 networks, for 2D grayscale and RGB images, all implemented in Python keras/tensorfow. The research was conducted also with the utilization of the Adaptive Vision platform, dedicated to engineering applications.

As one can see in Table 3, train accuracy for all methods, besides ResNet50, was over 96.9%, mostly close to 99.9% with very low train loss. For the test set, the results are slightly worse, which is mainly due to the fact, that none of the images from the test set is present in the training set. The training set will be enlarged in the future with hand gestures collected from more people with different complex backgrounds.

**Figure 19:**
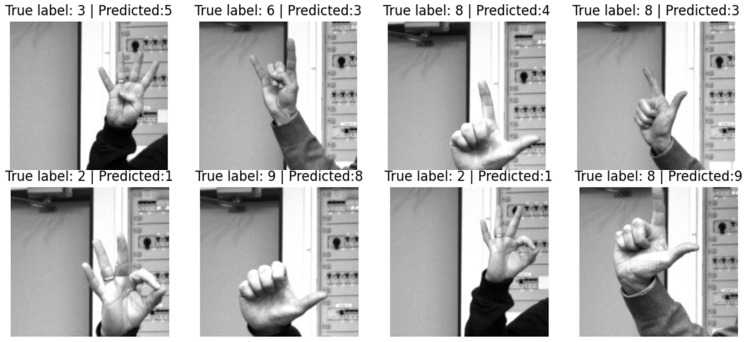
Examples of incorrectly classified gestures.

Some examples of incorrectly classified gestures are presented in Figure 19.

In addition, two approaches using 3D depth maps were also implemented—one model was trained in AVS, and the other in Python. The models showed a similar efficiency for the training set, but for the test set the AVS 3D network achieved almost 2% higher efficiency. The values of the loss function were also higher for CNN implemented in Python. The disadvantage of methods based on 2D images is the use ROI (the hand must appear in a specific area). The use of a 3D camera made it possible to provide the network with information on the distance of objects from the camera, while the images obtained with the 2D camera not. Thanks to this, the hand did not have to appear in a specific place (ROI), and the operator does not have to maintain a fixed location of the hand. Information about the distance is especially important when there is more than one person in the scene, then the gesture of the person closest to the camera is taken into account (which results from the recorded depth map). The second disadvantage faced by 2D vision is a complex background which has an impact on hand gesture detection and classification.

**Figure 20:**
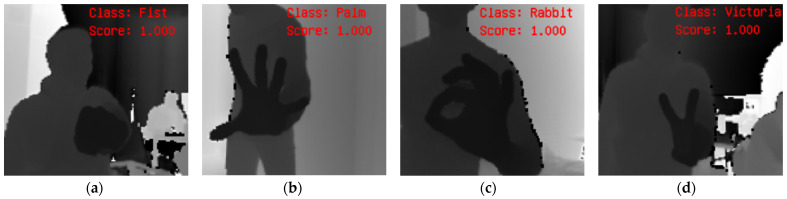
Fist(0) (**a**), Palm(1) (**b**), Rabbit(2) (**c**), Victoria(3) (**d**).

The AVS was utilized to capture the 3D images, generating depth images and manual classification to create a training set. Figure 20 presents exemplary results of gesture recognition in AVS for each gesture class. In the case of the test set, an accuracy of 99.60% was achieved (Table 4).

The average entropy of the training set is 0.004 and the test set is 0.019, which is much less than for 2D images. This method would be most suitable for use in industry, e.g., for AGV control or generally for unmanned ground vehicles, due to its flexibility and effective classification at the level of 99.60%. For comparison results, the CNN in Python was implemented once again. The CNN was exactly the same as in 2D vision described earlier. The accuracy of this model for the training set was 99.89% and for the test set 97.88%. The average entropy for the training set was 0.01 and for the test set 0.1. Initially, it seems that the classifier achieves higher accuracy than the 2D classifier with a smaller input image size. However, this requires detailed research in subsequent works, especially the expansion of the image database with further gestures.

## 4. Conclusions

The research shows that with a very limited set of sought features (in our case, specific types of hand gestures), it is enough to use simple networks with a small number of hidden layers. Complex networks, such as ResNet or Mobilenet, are trained on sets of images in which many features or objects are sought, such as animals, planes, people, cars, etc. Even training them, with transfer learning approach, to detect new objects will not always give the best result, and the classifier, with due to the size of these networks, it is relatively large.

For grayscale images, all networks gave relatively poor results, starting with CNN, which had the lowest accuracy for the test set, 70.53%, ending with ResNet50, which was 81.75% accurate. It is definitely better for RGB images, where apart from the ResNet50 network, the others oscillate with an accuracy of 90% for the test set, with the best result for the CNN network—90.53%.

Taking into account the fact, that the test difference in accuracy for the test set for different methods is not so big, in embedded solutions for AGV the method with lower sized classifier can be preferred.

The research results show that from the point of view of implementing gesture recognition methods in AGVs, the most promising are those using 2D RGB images and 3D imaging and a depth map. Due to the additional information, which is the distance of the object in 3D (the person showing the gesture) from the camera, it is easier to detect the hand and identify the gesture. In the initial stage of implementation, it will probably be necessary to narrow down the search area to ROI, due to the potentially complex background and proximity of other elements of the environment, i.e., objects between the person generating the gesture and the camera installed on the AGV. The person making the gesture will have to be in a free space—e.g., a passage in a production hall or warehouse, or make a gesture at an appropriate, defined height.

In the case of 2D RGB images, there is still place for improvement, namely expanding the 2D image database with a larger number of registered images within the gesture classes. The improvement of classification accuracy can be obtained at the initial stage by optimizing the ROI search algorithm. Currently, it is based on segmentation in the HSV space by searching for skin areas. In the future, we intend to improve this process by detecting body parts on the one hand—our database, as shown in the article, contains scenes with a full human pose and environment. On the other hand, the possibility of analyzing the scene and reducing the number of objects from outside the hand class in the background based on the YOLO object classifier. Naturally, this will simplify the scene and increase the chances of hand and palm area extraction.

## Figures and Tables

**Table 1:** Number of detected convexity defects for gestures 0–9.

	0	1	2	3	4	5
Fist(0)	459	16	0	0	0	0
Palm(1)	32	84	106	98	153	2
Rabbit(2)	153	179	122	21	0	0
Victoria(3)	37	356	81	1	0	0
One(4)	388	80	6	1	0	0
Four(5)	44	75	119	211	26	0
Rock(6)	117	337	21	0	0	0
Stop(7)	429	46	0	0	0	0
Loser(8)	247	216	11	1	0	0
Thumbleft(9)	387	87	1	0	0	0

**Table 2:** CNN architecture.

Layer Name	Output Size	Layer Type	Activation
Conv2D	240 × 240 × 16	Convolution	ReLU
MaxPooling2D	120 × 120 × 16	Max-Pooling	
Conv2D	116 × 116 × 32	Convolution	ReLU
MaxPooling2D	58 × 58 × 32	Max-Pooling	
Conv2D	54 × 54 × 64	Convolution	ReLU
MaxPooling2D	27 × 27 × 64	Max-Pooling	
Conv2D	23 × 23 × 128	Convolution	ReLU
MaxPooling2D	11 × 11 × 128	Max-Pooling	
Dense	64	Fully connected	ReLU
Dense	10	Fully connected	Softmax

**Table 3:** 2D CNN classifiers comparison.

Method	Train Accuracy [%]	Test Accuracy [%]	Train Loss	Test Loss	Classifier Size [MB]
Grayscale: AVS	99.90	78.90	0.022	0.332	14.00
Grayscale: MobileNetV2	97.44	78.37	0.087	0.312	42.40
Grayscale: ResNet50	91.98	81.75	0.237	0.496	196.00
Grayscale: Proposed CNN	99.93	70.53	0.004	1.719	14.40
RGB: AVS	100.00	89.80	0.015	0.124	14.00
RGB: MobileNetV2	96.94	90.13	0.087	0.312	42.40
RGB: ResNet50	94.47	85.00	0.168	0.438	196.00
RGB: Proposed CNN	99.90	90.53	0.004	0.403	14.50

**Table 4:** 3D CNN classifiers comparison.

Method	Train Accuracy [%]	Test Accuracy [%]	Train Loss	Test Loss	Classifier Size [MB]
AVS 3D	99.90	99.60	0.004	0.019	17.50
Proposed CNN 3D	99.89	97.88	0.010	0.100	4.32

## Data Availability

Not applicable.
